# Effectiveness and safety of rivaroxaban in patients with atrial fibrillation and heart failure in clinical practice: an indirect comparison of national and international registries

**DOI:** 10.3389/fcvm.2025.1451499

**Published:** 2025-05-27

**Authors:** Jose Maria Cepeda, Nicolas Manito, Alejandro Recio Mayoral, Iñaki Lekuona, Miguel Castillo Orive, Elvira Blanco Labrador, María Teresa Blasco, Nuria Farré, José Manuel García Pinilla, Javier Jiménez-Candil, Carles Rafols, Juan Jose Gomez Doblas

**Affiliations:** ^1^ Internal Medicine Department, Hospital Vega Baja, Orihuela, Spain; ^2^ Cardiology Department, Hospital Universitario de Bellvitge, Barcelona, Spain; ^3^ Cardiology Department, Hospital Universitario Virgen Macarena, Sevilla, Spain; ^4^ Cardiology Department, Hospital Galdakao-Usansolo, Bizkaia, Spain; ^5^ Cardiology Department, Hospital Universitario Ramón y Cajal, Madrid, Spain; ^6^ Cardiology Department, Complexo Hospitalario de Ourense, Ourense, Spain; ^7^ Cardiology Department, Hospital Universitario Miguel Servet, Zaragoza, Spain; ^8^ Cardiology Department, Hospital del Mar, Barcelona, Spain; ^9^ Cardiology Department, Hospital Clínico Universitario Virgen de la Victoria, Málaga, Spain; ^10^ IBIMA-Plataforma BIONAND, Ciber Cardiovascular, Instituto de Salud Carlos III, Departamento de Medicina y Dermatología, Universidad de Málaga, Málaga, Spain; ^11^ Cardiology Department, Hospital Universitario de Salamanca, Insitituto de Investigación Biomédica de Salamanca (IBSAL), Universidad de Salamanca, CIBER-CV, Salamanca, Spain; ^12^ Medical Department, Bayer Hispania, Barcelona, Spain; ^13^ Cardiology Department, Hospital Clínico Universitario Virgen de la Victoria, Málaga, Spain

**Keywords:** atrial fibrillation, anticoagulation, heart failure, rivaroxaban, clinical practice

## Abstract

**Background:**

The objective of the study was to analyze and compare the effectiveness and safety of rivaroxaban in patients with atrial fibrillation (AF) and heart failure (HF).

**Methods:**

The clinical profile and outcomes of the FARAONIC study were indirectly compared with those of the ROCKET-AF trial and other national and international observational registries.

**Results:**

In FARAONIC, the median age was 73.7 years, 34.1% were women, and the median CHA_2_DS_2_-VASc was 4.1. In the rivaroxaban arm of ROCKET-AF in patients with HF, these statistics were 72 years, 39.1%, and 5.1, respectively. In the national/international registries of patients with HF receiving rivaroxaban, these statistics were 74.0–75.3 years, 40.8%–41.4%, and 3.2–4.5, respectively. In the GLORIA-AF (dabigatran) and ETNA-AF (edoxaban) trials, these numbers were 69.9–75.3 years, 39.3%–41.6%, and 3.8–4.4, respectively. Among the HF populations, annualized rates of stroke or systemic embolism were 0.75% in FARAONIC (vs. 1.90% in ROCKET-AF, 0.92%–1.2% in national/international registries with rivaroxaban, 0.82% in GLORIA-AF, and 0.88% in ETNA-AF). Rates of major bleeding in FARAONIC were 1.55% (vs. 1.4%–3.86% in the national/international registries with rivaroxaban, 1.20% in GLORIA-AF, and 1.65% in ETNA-AF).

**Conclusion:**

In clinical practice, AF patients with HF, anticoagulated with rivaroxaban are old, have many comorbidities and have a high thromboembolic risk. Despite this, rates of adverse events are low.

## 1 Introduction

Heart failure (HF) and atrial fibrillation (AF) are two common cardiovascular conditions that frequently coexist (1–3). The prevalence of both conditions is increasing globally (4). AF can precipitate HF but it can also be a consequence of HF (1–3, 5). Patients with AF have a nearly fivefold increased risk of HF (6). In HF trials, the prevalence of AF ranges from 10% to 50% (7). Conversely, in clinical trials with direct oral anticoagulants (DOACs), among patients with AF, approximately 27%–65% of patients had HF concomitantly at baseline (8–11).

The concomitance of HF and AF markedly worsens the prognosis (12), and the risk of developing thromboembolic complications compared with the risk of each condition separately (1–3). Importantly, the stroke risk in patients with AF and HF is increased across the entire spectrum of left ventricular ejection fraction (3, 13). In this context, guidelines recommend chronic oral anticoagulation in these patients to reduce the risk of thromboembolic complications (14, 15).

**Figure 1:**
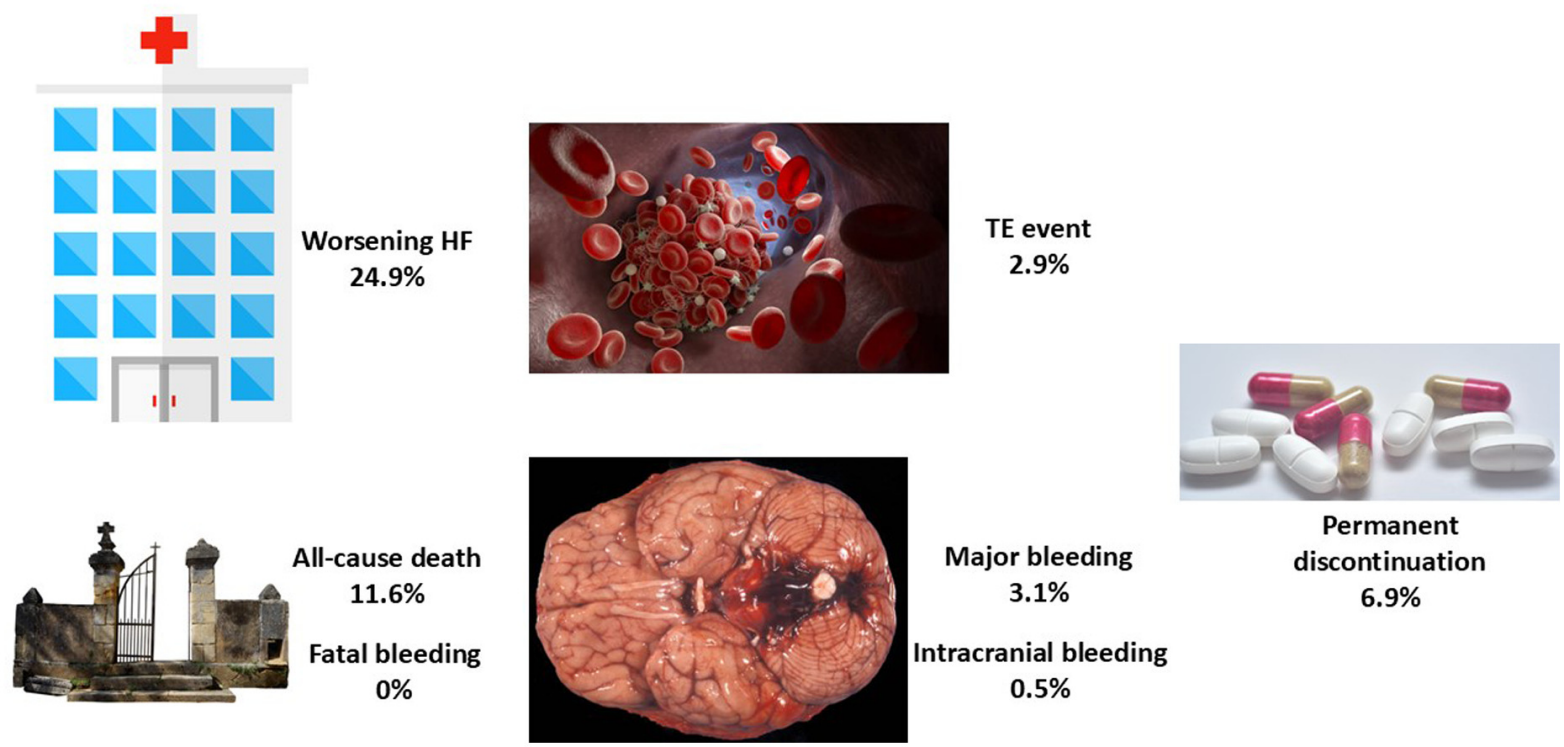
Main events in the FARAONIC registry after 2 years of follow-up. HF, heart failure; TE, thromboembolic event. Figure created with data from Manito et al. (18).

The ROCKET-AF trial showed that compared with warfarin, rivaroxaban was as effective for the prevention of stroke or systemic embolism, had a similar risk of major bleeding, and had a higher risk for major gastrointestinal bleeding, but had a significantly lower risk of intracranial and fatal hemorrhages (16). A specific substudy of the ROCKET-AF trial showed that the benefit of rivaroxaban was independent of HF status at baseline (8). Nevertheless, it is important to ascertain whether these results can be extended to real-life populations (17). The FARAONIC study was a prospective, multicenter, cohort study of patients with AF and HF, chronically treated with rivaroxaban, and aimed to determine the risk factors associated with worsening HF. This study showed that after 24 months of follow-up, approximately 25% of the patients developed HF worsening, and nearly 3% of the patients had a thromboembolic event and major bleeding, with a very low risk of intracranial bleeding and no cases of fatal hemorrhage (Figure 1) (18).

However, in the last few years, new clinical trials and a number of national and international studies on the use of rivaroxaban in clinical practice, according to HF status have been published (19–21). As a result, it is essential to ascertain whether the FARAONIC study results are comparable not only to the ROCKET-AF trial but also to other registries of rivaroxaban-treated patients. The aim of this article was to evaluate, through indirect comparisons between different national or international real-life studies, the clinical profile and outcomes of patients with AF and HF anticoagulated with rivaroxaban. In addition, data from the FARAONIC study were compared with two other observational and prospective studies of patients treated with other DOACs (22, 23).

## 2 Methods

FARAONIC was a Spanish multicenter, prospective, observational cohort study that included patients with non-valvular AF and chronic HF (regardless of ejection fraction) who received treatment with rivaroxaban for ≥4 months before being enrolled. A total of 672 patients from 71 Spanish centers were recruited, of whom 552 (82.1%) were included in the per-protocol analysis. In total, 51.3% of the patients had HF with preserved ejection fraction, 31.3% HF with reduced ejection fraction, and 17.4% HF with mildly reduced ejection fraction. Patients were followed up over a 2-year period (18).

First, to initially place the efficacy and safety of rivaroxaban in context, the results of phase III clinical trials with DOACs compared with warfarin according to baseline HF status were analyzed. ROCKET-AF was a double-blind clinical trial in which 14,264 patients with AF and a high stroke risk were randomized to rivaroxaban or warfarin. A total of 9,033 (63.7%) patients had HF. HF was defined as a history of HF or a left ventricular ejection fraction <40% (8). RE-LY was a clinical trial that compared two fixed and blinded doses of dabigatran (110 and 150 mg twice daily) with open-label dose-adjusted warfarin in 18,113 AF patients at increased risk for stroke, of whom 4,904 (27.1%) had HF at baseline. HF was defined as the presence of New York Heart Association (NYHA) class II–IV HF symptoms (fatigue and dyspnea) in the 6 months before being enrolled, in patients with a history of previous admission for congestive HF (9). ARISTOTLE was a double-blind randomized trial comparing apixaban with warfarin in patients with AF at risk of stroke. Information on investigator-reported HF and ventricular function was obtained from the trial case report forms and only patients with a report of both HF status and left ventricular function were included in this analysis. A total of 18,201 patients were initially randomized, of which 14,671 (81%) had information on both HF status and left ventricular systolic function. Of these, 18.6% had HF with reduced ejection fraction and 21.9% HF with preserved ejection fraction (10). ENGAGE AF-TIMI 48 was a randomized, double-blind clinical trial comparing edoxaban with warfarin. HF was defined as the presence or previous history of HF stage C or D according to the American College of Cardiology/American Heart Association (ACC/AHA) definition. Patients were classified as HF and NYHA classes I–II, HF and NYHA classes III–IV, and no HF. Patients in ACC/AHA stage B (asymptomatic left ventricular dysfunction) were considered as not having HF. Of the 14,071 patients randomized to warfarin or high-dose edoxaban, 5,926 (42%) had no history of HF at baseline, 6,344 (45%) mild HF, and 1,801 (13%) severe HF (11).

Second, the clinical profile and outcomes of the FARAONIC study were compared with those of two clinical trials (ROCKET-AF and AFIRE) and two national registries (EMIR and US database). In the AFIRE trial, Japanese patients with AF and stable coronary artery disease were randomized to receive rivaroxaban monotherapy or combination therapy with rivaroxaban and an antiplatelet agent. In this study, 2,215 patients were included; 36% (*n* = 788) had a history of HF (19). EMIR was a non-interventional and observational study that included adults with AF who had been administered rivaroxaban according to clinical practice for ≥6 months before being enrolled. Patients were recruited from 79 Spanish centers and followed up for 2.5 years. The information source was in all cases the medical record and the patient during the routine visits. A total of 1,433 patients were included in the final analysis, of whom 326 (22.7%) had HF at baseline (20). The US database was a retrospective claims database analysis of US Truven MarketScan data that combines two separate databases (a commercial and a Medicare supplemental database) from 1 November 2011 to 31 December 2016. For this study, patients with oral anticoagulant-naïve AF, HF, and ≥12 months of insurance coverage were identified. A total of 3,418 patients who received rivaroxaban were analyzed. Patients were followed up until an event, rivaroxaban discontinuation/switch, insurance disenrollment, or end of follow-up (21).

Finally, to put into context the results of rivaroxaban compared with those of other DOACs, two prospective registries were analyzed (GLORIA-AF and ETNA-AF). GLORIA-AF was a large, international, observational registry program that included patients with newly diagnosed AF at risk of stroke and CHA_2_DS_2_-VASc ≥1 in 44 countries from five geographical regions. Among the 4,873 dabigatran-treated patients, 1,169 (24.0%) had HF and 2-year outcomes were reported. HF was defined as NYHA classes II–IV or ejection fraction ≤40% (22). The Global ETNA-AF program included data from multiple prospective, observational, non-interventional regional studies of patients with AF receiving edoxaban for stroke prevention. Data from 27,333 patients, of whom 5,258 had HF history with 2-year annualized rates, were analyzed (23).

Whereas ROCKET-AF, RE-LY, ARISTOTLE, ENGAGE AF-TIMI 48, and AFIRE were randomized clinical trials, FARAONIC, EMIR, GLORIA-AF, and ETNA-AF were observational and prospective studies and the US database was an observational and retrospective study. Biodemographic data, comorbidities, NYHA functional class, HF treatments, and thromboembolic (CHADS_2_, CHA_2_DS_2_-VASc) and bleeding (HAS-BLED) risk were recorded if available. Adverse events, including stroke or systemic embolism, all-cause death, major bleeding, intracranial hemorrhage, and major adverse cardiovascular events (MACEs) were recorded from all studies if available. In addition, the proportion of patients that developed HF worsening (hospitalization or visit to the emergency department) and the proportion of permanent discontinuation of rivaroxaban during the follow-up in the FARAONIC study were also determined.

### 2.1 Statistical analysis

A descriptive analysis was performed and data were compared numerically (indirect comparisons). Quantitative variables were reported with mean or median, as available, and qualitative variables as relative frequencies (percentages). Events were recorded from the original publication of the clinical trials and registries, including stroke or systemic embolism, all-cause death, major bleeding, intracranial bleeding, and MACEs, when available. The annual incidence of events was calculated in the FARAONIC study and annual rates expressed as events per 100 patient-years were recorded for the rest of studies from the original publications. The data were analyzed using the statistical package SPSS (v18.0 or superior).

## 3 Results

In the FARAONIC study, at baseline, the mean age was 73.7 ± 10.9 years, 65.9% were men, and 33.9% were considered frail. With regard to AF, 53.9% of patients had permanent AF, CHA_2_DS_2_-VASc was 4.1 ± 1.5, and HAS-BLED was 1.6 ± 0.9. Comorbidities were common, as 77.5% had arterial hypertension, 39.1% had previous coronary artery disease, 37.3% had diabetes, and 32.4% had chronic kidney disease. Furthermore, 51.3% of the patients had HF with preserved ejection fraction. Regarding HF treatments at baseline, 90.6% received diuretics, 85.5% a renin-angiotensin system inhibitor (36.7% received an angiotensin-converting enzyme inhibitor, 23.8% an angiotensin II receptor blocker, and 25.0% sacubitril/valsartan), 79.7% a beta blocker, 51.4% an aldosterone antagonist, 23.0% digoxin, and 3.1% ivabradine. After 24 months of follow-up, 11.6% of the patients had died, 2.9% had a thromboembolic event, 3.1% had a major bleeding event, 0.5% had an intracranial bleeding event, and no patient had a fatal hemorrhage (18).

### 3.1 ROCKET-AF, RE-LY, ARISTOTLE, and ENGAGE AF-TIMI 48 trials, according to baseline HF status

In ROCKET-AF, the patients with HF were younger and had higher levels of hypertension, diabetes, history of myocardial infarction, and thromboembolic risk, but less prior cerebrovascular disease compared to those patients without HF (8). In RE-LY, the patients with HF were younger and had less prior history of cerebrovascular disease and hypertension, but higher levels of diabetes, history of coronary artery disease, and a higher thromboembolic risk (9). In ARISTOTLE, the HF population was younger and had less history of cerebrovascular disease, but higher levels of ischemic heart disease and thromboembolic risk (10). In ENGAGE AF-TIMI 48, the patients with HF were younger and less likely to have prior cerebrovascular disease and diabetes. However, the patients with HF were more likely to have hypertension and had a higher thromboembolic risk (11) (Table 1).

**Table 1:** Clinical characteristics of the patients included in the ROCKET-AF, RE-LY, ARISTOTLE, and ENGAGE AF-TIMI 48 trials, according to baseline HF status.

Clinical characteristics	ROCKET-AF (rivaroxaban arm)		RE-LY (dabigatran 110 arm)		RE-LY (dabigatran 150 arm)		ARISTOTLE (overall)			ENGAGE AF-TIMI 48 (overall)	
	HF (*n* = 4,530; 64.0%)	No HF (*n* = 2,551; 36.0%)	HF (*n* = 1,641; 27.3%)	No HF (*n* = 4,374; 72.7%)	HF (*n* = 1,640; 27.0%)	No HF (*n* = 4,436; 73.0%)	HFrEF (*n* = 2,736; 18.6%)	HFpEF (*n* = 3,207; 21.9%)	No HF (*n* = 8,728; 59.5%)	HF (*n* = 8,145; 58%)	No HF (*n* = 5,926; 42%)
Age, years	72	74	68.5	72.5	68	72.8	68	69	71	70	75
Female, %	39.1	40.3	30.4	37.7	35.3	37.4	21	42	35	37.4	38.1
Stroke or TIA^a^, %	42.9	70.0	16.5^a^	23.7^a^	18.7^a^	23.7^a^	16	17	20	21.2	37.9
CHADS_2_	3.7	3.2	2.6	2.0	2.7	2.0	2.2	2.7	1.9	3.0	2.6
CHA_2_DS_2_-VASc	5.1	4.5	NR	NR	NR	NR	NR	NR	NR	4.5	4.1
LVEF <40%^b^, %	33.3	0	44.0	11.2	44.0	11.1	86	0	0	49.0^b^	10.1^b^
NYHA class, %		NR	NR	NR	NR	NR				NYHA I–II: 77.9 NYHA III–IV: 22.1	NR
I	13.7						27	16	73		
II	56.4						50	62	24		
III	28.3						22	21	2		
IV	1.7						1	<1	<1		
Hypertension, %	92.8	85.7	74.9	80.2	75.0	80.4	75	89	90	94.0	93.1
Diabetes, %	42.3	36.7	25.6	22.6	27.9	21.3	27	25	25	30.4	43.9
Coronary artery disease, %	NR	NR	31.8	26.0	31.4	26.9	43	48	29	NR	NR
Myocardial infarction, %	20.8	9.1	NR	NR	NR	NR	28	18	11	14.3	7.7
Previous VKA use, %	58.7	68.8	NR	NR	NR	NR	61	51	63	43.5	38.0
Beta blockers, %	68.7	56.8	68.1	61.1	70.4	61.6	75	69	62	71.1	59.4
Digitalis, %	44.7	27.4	NR	NR	NR	NR	47	39	24	NR	NR
ACEi, %	61.6	41.5	57.2	40.2	58.7	40.4	81	77	66	71.0	58.7
ARB	NR	NR	21.8	24.9	21.5	25.2					
Diuretics, %	71.4	39.4	72.0	42.8	72.5	43.5	73	70	46	72.1	43.7

NR, not reported; ACEi, angiotensin-converting enzyme inhibitor; ARB, angiotensin receptor blocker; HF, heart failure; LVEF, left ventricular ejection fraction; NYHA, New York Heart Association; TIA, transient ischemic attack; VKA, vitamin K antagonists.

^a^ Stroke or TIA or systemic embolism.

^b^ <50%.

Table created with data from references (8–11).

The median follow-up in the four clinical trials ranged from 1.5 to 2.8 years. Among DOAC arms of the clinical trials, the annualized event rates for stroke or systemic embolism ranged from 0.99% to 1.90% in the HF population (vs. from 1.0% to 2.1% in the non-HF population). Annualized event rates for major bleeding were 1.95%–3.26% and 2.17%–3.39%, for HF and non-HF patients, respectively. Annualized event rates for intracranial bleeding were 0.15%–0.40% and 0.23%–0.64%, for HF and non-HF patients, respectively. Annualized event rates for all-cause death were 4.36%–6.99% and 2.17%–3.20%, for HF and non-HF patients, respectively (Table 2).

**Table 2:** Adverse events^a^ in the ROCKET-AF, RE-LY, ARISTOTLE, and ENGAGE AF-TIMI 48 trials, according to baseline HF status.

Adverse events	ROCKET-AF (rivaroxaban arm)		RE-LY (dabigatran 110 arm)		RE-LY (dabigatran 150 arm)		ARISTOTLE (apixaban)			ENGAGE AF-TIMI 48 (edoxaban)	
	HF (*n* = 4,530; 64.0%)	No HF (*n* = 2,551; 36.0%)	HF (*n* = 1,641; 27.3%)	No HF (*n* = 4,374; 72.7%)	HF (*n* = 1,640; 27.0%)	No HF (*n* = 4,436; 73.0%)	HFrEF	HFpEF	No HF	HF (*n* = 8,145; 58%)	No HF (*n* = 5,926; 42%)
Median follow-up, years	1.9		2.0		2.0		1.5			2.8	
Stroke or SE	1.90	2.10	1.90	1.41	1.44	1.00	0.99	1.51	1.16	NYHA I–II: 1.52 NYHA III–IV: 1.83	1.54
All-cause death	5.05	3.20	NR	NR	NR	NR	6.99	4.05	2.17	NYHA I–II: 4.36 NYHA III–IV: 6.50	2.89
Major bleeding	14.22^b^	16.12^b^	3.26	2.73	3.10	3.39	2.77	1.95	2.17	NYHA I–II: 2.61 NYHA III–IV: 2.49	2.98
Intracranial hemorrhage	0.40	0.64	0.22	0.23	0.26	0.34	0.18	0.15	0.38	NYHA I–II: 0.35 NYHA III–IV: 0.32	0.46

NR, not reported; NYHA, New York Heart Association.

^a^ Reported as events per 100 patient-years.

^b^ Major or clinically relevant non-major bleeding.

Table created with data from references (8–11).

### 3.2 ROCKET-AF trial and FARAONIC, AFIRE, EMIR, and US database registries, according to baseline HF status

The clinical characteristics of the patients with AF treated with rivaroxaban in the ROCKET-AF trial and different registries, according to baseline HF status, are presented in Table 3. In the patients with HF, the median age of the patients included in the registries (73.7–75.3 years) was higher than that of the ROCKET-AF trial (72 years). The HF population included in the registries had fewer comorbidities than the patients included in the ROCKET-AF trial and lower thromboembolic risk (CHA_2_DS_2_-VASc 3.9–4.5 vs. 5.1, respectively). There were some differences in the proportion of patients treated with HF drugs depending on the registries. With regard to clinical events in the HF population, annualized rates/incidence of stroke or systemic embolism were 0.75%–0.98% in the registries (vs. 1.90% in the ROCKET-AF trial). These rates for major bleeding, intracranial bleeding, and all-cause death were 1.4%–3.86% vs. 14.22% (including major or clinically relevant non-major bleeding), 0.25%–0.27% vs. 0.40%, and 3.14%–5.8% vs. 5.05%, respectively (Table 4) (8, 18–21).

**Table 3:** Clinical characteristics of the patients with AF treated with rivaroxaban in the ROCKET-AF trial and the FARAONIC, AFIRE, EMIR, and US database registries, according to baseline HF status.

Clinical characteristics	ROCKET-AF (rivaroxaban arm)		FARAONIC (rivaroxaban)	AFIRE (monotherapy arm)		EMIR (rivaroxaban)		US database (rivaroxaban)
	HF (*n* = 4,530; 64.0%)	No HF (*n* = 2,551; 36.0%)	HF (*n* = 552)	HF (*n* = 389; 35.5%)	No HF (718; 64.5%)	HF (*n* = 326; 22.7%)	No HF (*n* = 1,107; 77.3%)	HF (*n* = 3,418; 100%)
Age, years	72	74	73.7	75.3	73.8	75.3	73.8	74
Female, %	39.1	40.3	34.1	NR	NR	40.8	53.2	41.4
Stroke or TIA^a^, %	42.9	70.0	12.5	NR	NR	12.9	12.4	7.7^a^
CHADS2	3.7	3.2	NR	NR	NR	NR	NR	NR
CHA2DS2-VASc	5.1	4.5	4.1	NR	NR	4.5	3.2	3.9
LVEF <40%, %	33.3	0	31.3	NR	NR	28.8	0	NR
NYHA class, %		NR		NR	NR	NR	NR	NR
I	13.7		17.4					
II	56.4		58.7					
III	28.3		23.2					
IV	1.7		0.7					
Hypertension, %	92.8	85.7	77.5	NR	NR	80.1	79.1	82.9
Diabetes, %	42.3	36.7	37.3	NR	NR	36.5	24.3	35.2
CAD	NR	NR	39.1	100	100	28.2	12.9	NR
Myocardial infarction, %	20.8	9.1	NR	—		14.4	NR	13.2
Previous VKA use, %	58.7	68.8	44.9	NR	NR	NR	NR	NR
β-Blocker, %	68.7	56.8	79.7	NR	NR	NR	NR	64.5
Digitalis, %	44.7	27.4	23.0	NR	NR	NR	NR	11.1
ACEi, %	61.6	41.5	85.5	NR	NR	NR	NR	61.6
ARB	NR	NR						
Diuretics, %	71.4	39.4	90.6	NR	NR	NR	NR	72.8

NR, not reported; ACEi, angiotensin-converting enzyme inhibitor; ARB, angiotensin receptor blocker; HF, heart failure; LVEF, left ventricular ejection fraction; NYHA, New York Heart Association; TIA, transient ischemic attack; VKA, vitamin K antagonists; CAD, coronary artery disease.

^a^ Ischemic stroke.

Table created with data from references (8, 18–21).

**Table 4:** Adverse events^a^ of the patients with AF treated with rivaroxaban in the ROCKET-AF trial and the FARAONIC, AFIRE, EMIR, and US database registries, according to baseline HF status.

Adverse events	ROCKET-AF (rivaroxaban arm)		FARAONIC (rivaroxaban)	AFIRE (monotherapy arm)		EMIR (rivaroxaban)		US database (rivaroxaban)
	HF (*n* = 4,530; 64.0%)	No HF (*n* = 2,551; 36.0%)	HF (*n* = 552)	HF (*n* = 389; 35.5%)	No HF (718; 64.5%)	HF (*n* = 326; 22.7%)	No HF (*n* = 1,107; 77.3%)	HF (*n* = 3,418; 100%)
Median follow-up, years	1.9		2.0	2.0		2.5		1.4
Stroke or SE^b^^,^^d^	1.90	2.10	0.75	0.92^b^	0.98^b^	1.2^d^	0.6^d^	0.98
All-cause death	5.05	3.20	5.8	3.14	1.17	5.5	2.0	NR
Major bleeding	14.22^c^	16.12^c^	1.55	1.60	1.62	1.4	0.9	3.86
Intracranial hemorrhage	0.40	0.64	0.25	NR	NR	NR	NR	0.27
MACE	NR	NR	NR	3.89	2.25	3.0	0.5	NR

NR, not reported; MACE, major adverse cardiac event.

^a^ Annual incidence of events was calculated in the FARAONIC study and annual rates expressed as events per 100 patient-years were recorded for the rest of the studies.

^b^ Ischemic stroke.

^c^ Major or clinically relevant non-major bleeding.

^d^ Stroke + SE + TIA; SE: systemic embolism.

Table created with data from references (8, 18–21).

### 3.3 FARAONIC, GLORIA-AF, and ETNA-AF registries

Three registries with different DOACs (FARAONIC, rivaroxaban; GLORIA-AF, dabigatran; and ETNA-AF, edoxaban) were analyzed to determine the clinical profile and outcomes in the HF population (Tables 5, 6). The patients with HF included in these registries were old (age 69.9–75.3 years), 10.1%–13.0% had prior cerebrovascular disease, 29.8%–39.1% had coronary artery disease, and 23.9%–37.3% had diabetes and a high thromboembolic risk (CHA_2_DS_2_-VASc 3.8–4.4). In all registries, a high proportion of patients were receiving HF drugs. The follow-up or outcomes were similar in the three registries (2 years). In the HF population in GLORIA-AF and ETNA-AF, annualized rates for stroke or systemic embolism, major bleeding, intracranial bleeding, and all-cause death were 0.75%–0.88%, 1.20%–1.65%, 0.25%–0.36%, and 4.76%–6.08%, respectively. With regard to the dose of DOACs used in each registry in the HF population, in FARAONIC, 69% of patients were receiving rivaroxaban 20 mg (31% rivaroxaban 15 mg); in GLORIA-AF, 50.1% were receiving dabigatran 150 mg, 47.3% dabigatran 110 mg, and 2.0% dabigatran 75 mg; and in ETNA, AF 36.9% were receiving edoxaban 60 mg and 63.1% edoxaban 30 mg (18, 22, 23).

**Table 5:** Clinical characteristics of the patients with AF included in the FARAONIC (rivaroxaban), GLORIA-AF (dabigatran), and ETNA-AF (edoxaban) registries, according to baseline HF status.

Clinical characteristics	FARAONIC (rivaroxaban)	GLORIA-AF (dabigatran)		ETNA-AF (edoxaban)	
	HF (*n* = 552)	HF (*n* = 1,169; 24.2%)	No HF (*n* = 3,658;75.8%)	HF (*n* = 5,258; 19.2%)	No HF (*n* = 22,075;80.8%)
Age, years	73.7	69.9	70.3	75.3	73.3
Female, %	34.1	39.3	46.0	41.6	41.9
Stroke or TIA^a^, %	12.5	10.1	17.5	13.0^a^	11.5^a^
CHADS2	NR	NR	NR	NR	NR
CHA2DS2-VASc	4.1	3.8	3.0	4.4	3.0
LVEF <40%, %	31.3	38.2	0	NR	NR
NYHA class, %			NR	NR	NR
I	17.4	9.6			
II	58.7	50.6			
III	23.2	24.7			
IV	0.7	4.6^+^			
Hypertension, %	77.5	76.8	77.7	76.7	73.5
Diabetes, %	37.3	23.9	22.4	29.6	21.7
CAD	39.1	29.8	15.5	NR	NR
Myocardial infarction, %	NR	15.2	6.6	9.1	2.5
Previous VKA use, %	44.9	NR	NR	NR	NR
β-blocker, %	79.7	70.9	60.2	NR	NR
Digitalis, %	23.0	19.9	7.2	NR	NR
ACEi, %	85.5	45.9	29.3	NR	NR
ARB		25.5	28.9		
Diuretics, %	90.6	66.6	31.3	NR	NR

NR, not reported; ACEi, angiotensin-converting enzyme inhibitor; ARB, angiotensin receptor blocker; HF, heart failure; LVEF, left ventricular ejection fraction; NYHA, New York Heart Association; TIA, transient ischemic attack; VKA, vitamin K antagonists; CAD, coronary artery disease.

^a^ Ischemic stroke.

^+^For 386 patients (10.5%), NYHA class was unknown.

Table created with data from references (18, 22, 23).

**Table 6:** Adverse events^a^ of the patients with AF included in the FARAONIC (rivaroxaban), GLORIA-AF (dabigatran), and ETNA-AF (edoxaban) registries, according to baseline HF status.

Adverse events	FARAONIC (rivaroxaban)	GLORIA-AF (dabigatran)		ETNA-AF (edoxaban)	
	HF (*n* = 552)	HF (*n* = 1,169; 24.2%)	No HF (*n* = 3,658; 75.8%)	HF (*n* = 5,258; 19.2%)	No HF (*n* = 22,075; 80.8%)
Median follow-up, years	2.0	2.0		2.0	
Stroke or SE	0.75	0.82^b^	0.60^b^	0.88^c^	0.71^c^
All-cause death	5.8	4.76	1.80	6.08	2.52
Major bleeding	1.55	1.20	0.92	1.65	0.88
Intracranial hemorrhage	0.25	NR	NR	0.36	0.27
MACE	NR	NR	NR	NR	NR

SE, systemic embolism; NR, not reported; MACE, major adverse cardiac event.

^a^ Annual incidence of events was calculated in the FARAONIC study and annual rates expressed as events per 100 patient-years were recorded for the rest of the studies.

^b^ Stroke.

^c^ Ischemic stroke.

Table created with data from references (18, 22, 23).

## 4 Discussion

The FARAONIC study showed in a diverse sample of real-life patients with AF and HF anticoagulated with rivaroxaban that these patients were old, had many comorbidities, and had a high thromboembolic risk. Despite this, the rates of thromboembolism, death, and major bleeding remained low. These numbers were lower than those reported in ROCKET-AF and other clinical trials with DOACs but in line with other national or international registries.

Although the most important complication in patients with AF is the development of ischemic stroke that is associated with great morbidity and mortality (24), anticoagulated patients with AF have a substantial residual risk of other outcomes, such as mortality or MACEs. This is even more important in patients with concomitant HF (1–3). In this context, the optimal treatment strategies for patients with HF and AF remain unclear. Traditionally, in recent decades, vitamin K antagonists have been used to reduce the risk of stroke in AF patients with HF. However, they have many limitations that increase in HF, in which suboptimal levels of warfarin anticoagulation control are more common, leading to more complications (25). However, a meta-analysis of four clinical trials that compared DOACs and warfarin showed that overall, DOACs were more effective, with a lower risk of death and bleeding (26). For these reasons, it is crucial to ascertain whether the benefits of DOACs over warfarin remain in the HF population. In our study, we analyzed the clinical profile and clinical outcomes of four phase III clinical trials with DOACs. Despite the worse clinical profile and higher thromboembolic risk of patients with HF, clinical trials showed that the relatively better efficacy and safety of DOACs over warfarin persisted in the HF population (8–11, 27), suggesting that the use of DOACs should be preferred over vitamin K antagonists in this population (1–3, 14, 15). In fact, a recent meta-analysis showed that in patients with HF and AF, compared with warfarin, DOACs significantly reduced the risk of stroke or systemic embolism by 17%, all-cause mortality by 15%, major bleeding by 11%, and intracranial hemorrhage by 46%. These beneficial effects extended to the overall spectrum of patients with HF (28). In summary, oral anticoagulation should be recommended for all patients with AF and HF, independent of HF type, making DOAC the first choice in this population (29).

Compared with the rivaroxaban arm of the ROCKET-AF trial, patients included in the FARAONIC study had a better clinical profile, with a lower proportion of comorbidities and thromboembolic risk (8, 18). This is related to the ROCKET-AF inclusion criteria that represented the more advanced clinical scenario of patients with AF included in all phase III clinical trials with DOACs (8–11). By contrast, the FARAONIC study included patients with AF and HF who were representative of clinical practice, as no strict selection criteria were defined. In fact, the clinical profile of the FARAONIC study was similar to that of other national registries including patients with HF and AF, anticoagulated with rivaroxaban (18, 20, 21). In the registries of patients who received rivaroxaban, annual rates of stroke or systemic embolism were low (0.75%–0.98% vs. 1.90% in the ROCKET-AF trial), suggesting that rivaroxaban was effective in the treatment of this very high-risk population in clinical practice, with a low risk of bleeding, particularly intracranial hemorrhage (18, 20, 21). In addition, no cases of fatal bleeding were observed in FARAONIC (18).

Furthermore, the EMIR study showed that HF was independently associated with the development of MACEs, but not with thromboembolic or bleeding events (20). This means that despite proper anticoagulation, patients with AF and HF still have a residual risk of developing cardiovascular complications. The optimal management of patients with AF and HF should not be limited to anticoagulation but include a comprehensive therapeutic approach (14, 15). In this context, choosing the most appropriate oral anticoagulant is mandatory. Thus, experimental data in a mouse model have shown that rivaroxaban may suppress the progression of ischemic cardiomyopathy and reduce cardiac dysfunction of ischemic origin and clinical studies have suggested that rivaroxaban could reduce the risk of myocardial infarction and cardiovascular death in the AF population (30–32).

In addition, we compared the clinical profile and rates of adverse events of the FARAONIC study with some international clinical registries with other DOACs, i.e., GLORIA-AF (dabigatran) and ETNA-AF (edoxaban) (18, 22, 23). Although only indirect comparisons can be performed, it seems that despite a similar clinical profile, the incidence of stroke or systemic embolism was lower in the FARAONIC study, with a similar risk of bleeding. This could be related to the higher use of reduced DOAC doses reported in the GLORIA-AF and ETNA-AF registries compared with the FARAONIC study, which may lead to reduced effectiveness in clinical practice (33). This could be explained by the fact that the GLORIA-AF and ETNA-AF registries started earlier, and physicians were not as confident with the use of DOACs as in the FARAONIC study. In addition, in the FARAONIC study, persistence with rivaroxaban was very high (permanent discontinuation of 6.9%) after 24 months of follow-up (18). This is important because this indicates not only the good tolerability of rivaroxaban but also because medication persistence is crucial to assure good efficacy in patients with chronic conditions. In addition, the once-daily dose of rivaroxaban could also enhance medication adherence, particularly in this polymedicated population (34).

HF treatments were underused in the FARAONIC study and in the other registries. However, it should be noted that 51.3% of patients included in the FARAONIC study had HF with preserved ejection fraction and at the moment of recruitment (between March 2018 and July 2019), no specific drugs had been approved for this indication (18). Regardless, this type of study clearly indicates that more efforts should be made to improve the management of our patients, not only from an HF perspective but with a holistic approach, treating all the comorbidities (14, 15).

This study has some limitations. Due to the design of this study, only indirect comparisons between the studies were made and these were descriptive and non-adjusted. As a result, no definite conclusions can be obtained from these comparisons and no more than hypotheses can be suggested. In this context, these data should be confirmed in further studies, with longer follow-ups. However, observational studies are the best design to reflect clinical practice. In addition, the high number of patients included in the national and international registries may reduce these potential biases, providing relevant information about a population that has not been well characterized.[Table T7]

**Table T7:** 

Center	Principal researcher
Hospital del Mar	Nuria Farré López
Hospital Universitario Lucus Augusti	Margarita Regueiro Abel
Hospital Basurto	Ainara Lozano Bahamonde
Consulta Privada Dr. Torres	Francisco Torres Calvo
Complejo Hospitalario de Santiago	Rosa María Agra Bermejo
Clínica Cardiología Vera	Eduardo Sebastián López Sánchez
Consulta Cardiológica Ricardo Fajardo Molina	Ricardo Fajardo Molina
CHOU Ourense	Gloria López Barros
Hospital de Galdakao/Usansolo	Mª Angeles Eneriz
Hospital Universitario Ramón y Cajal	Susana del Prado
Complejo Hospitalario de Navarra	Ana Carmen Abecia Ozcariz
Consorci Sanitario de Terrassa	Joan Martinez Tur
Complejo Hospitalario de Ferrol (H. Arquitecto Marcide)	Manuel López Pérez
Hospital Regional de Málaga Carlos Haya	José María Pérez Ruiz
Hospital Virgen de la Victoria	Jose Manuel Garcia Pinilla
Hospital Universitari de Girona Doctor Josep Trueta	Julia Roure Fernandez
Hospital Rey Juan Carlos I de Móstoles	Elena Mejia Martinez
Hospital Rio Hortega de Valladolid	Mª del Mar de la Torre Carpente
Consulta Dr. Enrique Galve Basilio	Enrique Galve Basilio
Hospital Doce de Octubre	Daniel Ferreiro
Cardioempordà	Sara Darnés Soler
Hospital Clínico Universitario de Valladolid	Pedro Ángel de Santos Castro
Hospital Virgen de las Nieves	Silvia López-Fernández
Hospital Puerto Real	Fco. Javier Camacho Jurado
Hospital Universitario San Cecilio	Jesús Gabriel Sanchez Ramos
Hospital La Paz	Isabel Antorrena
Hospital Universitario Donostia	Irene Rilo Miranda
Hospital Puerta del Mar	Daniel Bartolome Mateos
Hospital San Carlos	Francisco Manuel Brun Romero
Hospital Clínico Universitario de Salamanca	Elisabete Alzola Martinez
Complejo Asist. Univ. León	José Ignacio Iglesias Garriz
Hospital Costa de la Luz	María Rosario Perez Tristancho
Hospital de Burgos	Esther Sánchez Corral
Hospital Rio Carrión (Complejo Aistencial Universitario)	Jose Ignacio Cuende Melero
Hospital Comarcal Monforte de Lemos	Ricardo Izquierdo
Clínica Clivina	María Rosa Fernández Olmo
Complejo Asistencial de Soria (Hospital Santa Barbara)	Margarita Carrera Izquierdo
Fundación Hayge	Pere Álvarez García
Hospital Poniente	Juan A. Montes Romero
Hospital Universitario La Zarzuela (Sanitas)	Santiago de Dios
Hospital Virgen Macarena	Alejandro Recio Mayoral
Complejo Hospitalario de Pontevedra (Hospital de Montecelo)	Juan Carlos Rodríguez García
Hospital de Sierrallana	Pilar Ortiz Oficialdegui
Hospital Clínic i Provincial	Ana García Alvarez
Hospital Clínico Universitario Lozano Blesa	Juan Ignacio Perez Calvo
Hospital Miguel Servet	Ana Portoles Ocampo
Hospital Royo Vilanova	David Bierge Valero
Hospital Sanchinarro	Francisco Javier Parra
Hospital Monteprincipe	Francisco J. Rodriguez Rodrigo
Hospital Sant Pau	Sonia Mirabet Perez
Hospital Arrixaca	Domingo Pascual Figal
Hospital Morales Meseguer	Diego Miguel Giménez Cervantes
Hospital Moises Broggi	Roman Freixa Pamias
Hospital de Cruces	Ángel Sebastián Leza
Hospital de Bellvitge	Josep Comin Colet
Hospital Infanta Leonor de Madrid	David Vaqueriza Cubillo
Hospital Nuestra Señora de Sonsoles	Rosa Ana Lopez Jiménez
Hospital del Sagrat Cor	Martin Luis Descalzo
Hospital Sant Joan de Déu de Martorell	María Ysabel Saldarriaga Infante
Complejo Hospitalario Ruber Juan Bravo	María Carmen Gómez Rubín
Hospital Universitari Germans Trias i Pujol	Javier Santesmases Ejarque
Hospital de la Princesa	Berta Moyano
Hospital Universitari Vall d'Hebron	Teresa Soriano Sanchez
Hospital General San Jorge	Maria Teresa Villarroel Salcedo
Hospital Infanta Sofía	Diego Iglesias Del Valle
Hospital Virgen de la Luz	José Antonio Nieto Rodriguez
Centro Médico Lamar	Monzer Khanji Khatib
Clínica Nuestra Señora del Rosario	Maria Carmen Alonso Gutierrez
Hospital San Rafael	Gonzalo Peña Pérez
Hospital Povisa	Fernando Soto Loureiro

## 5 Conclusion

In conclusion, in clinical practice, patients with AF and HF, anticoagulated with rivaroxaban, are old, have many comorbidities, and have a high thromboembolic risk. Despite this, the rates of adverse outcomes, including stroke, all-cause death, and bleeding, are low.

## Data Availability

The raw data supporting the conclusions of this article will be made available by the authors, without undue reservation.
